# Uncommon Complications of Osteomyelitis: Deep Vein Thrombosis and Pulmonary Septic Emboli in Two Healthy Pediatric Patients: A Case Report and Literature Review

**DOI:** 10.1155/crpe/3736016

**Published:** 2026-04-30

**Authors:** Hala O. Abdallah, Lara Haj Mohammed, Hobab Jehad Odeh, Alma Abu Lil, Amjad Rajab

**Affiliations:** ^1^ Faculty of Medicine Department, An-Najah National University, Nablus, State of Palestine, najah.edu; ^2^ Faculty of Medicine Department, Alexandria University, Alexandria, Egypt, alexu.edu.eg; ^3^ Faculty of Medicine Department, Jordan University of Science and Technology, Irbid, Jordan, just.edu.jo; ^4^ Pediatric Department, Istishari Arab Hospital, Ramallah, State of Palestine

**Keywords:** deep vein thrombosis, osteomyelitis, pediatrics, pulmonary septic emboli

## Abstract

Acute osteomyelitis in children is typically a localized hematogenous infection; however, its presentation with deep vein thrombosis (DVT) and septic pulmonary emboli (SPE) is exceptionally rare. Fewer than fifteen similar cases have been reported in the literature. We describe two previously healthy pediatric patients who developed multifocal osteomyelitis, DVT, and SPE in the setting of methicillin‐resistant *Staphylococcus aureus* (MRSA) bacteremia. Both children initially presented with nonspecific limb pain, fever, and markedly elevated inflammatory markers, with early imaging proving nondiagnostic. Each subsequently developed venous thrombosis and septic emboli prior to confirmation of osteomyelitis, reflecting an aggressive clinical pattern. MRI later revealed multifocal bone involvement with subperiosteal abscesses requiring surgical drainage. Management required prolonged targeted intravenous antibiotics, anticoagulation, and coordinated multidisciplinary care. These cases highlight the importance of early suspicion for musculoskeletal infection in febrile children with persistent limb pain and systemic MRSA infection. Early MRI and prompt recognition of thromboembolic complications are essential for improving outcomes.

## 1. Introduction

Osteomyelitis is a bacterial infection of bone, most frequently affecting the long bones in children and adolescents, owing to their rich metaphyseal blood supply [1]. While most cases of acute hematogenous osteomyelitis in pediatric populations are successfully managed with prompt antimicrobial therapy, often targeting *Staphylococcus aureus*, the disease can sometimes evolve in unpredictable ways [2]. Although uncommon, vascular complications such as deep vein thrombosis (DVT) and septic pulmonary emboli (SPE) have been reported in association with musculoskeletal infections including osteomyelitis [3, 4]. The rarity of these complications in otherwise healthy pediatric patients makes them easily overlooked, thereby delaying diagnosis and treatment. A comprehensive review focusing on musculoskeletal infection–associated DVT found that osteomyelitis accounted for the majority of cases and that pulmonary involvement, presumably due to septic emboli, occurred in a substantial portion [2].

The coexistence of osteomyelitis, DVT, and SPE represents a diagnostic and therapeutic challenge. In many cases, early clinical presentation may be dominated by bone pain, fever, and localized signs of infection, overshadowing vascular or pulmonary complications. Consequently, awareness of such rare but serious sequelae is critical for clinicians managing pediatric bone infections.

In this context, we present two cases of previously healthy pediatric patients who developed DVT and pulmonary septic emboli in the setting of acute osteomyelitis. Through these cases, we aim to [1] underscore the potential for severe vascular and pulmonary complications even in healthy children with osteomyelitis [2], illustrate the clinical presentation, diagnostic workup, and challenges associated with identifying DVT and SPE in this population, and [3] review the existing literature to inform clinical suspicion and management strategies. By raising awareness of these uncommon but life‐threatening complications, we hope to contribute to improved recognition, timely diagnosis, and optimized management of osteomyelitis in pediatric patients.

## 2. Case Presentation

### 2.1. Case #1

An 11‐year‐old male, previously healthy and fully immune, presented with a 4‐day history of undocumented fever and left wrist pain after a minor trauma involving the left wrist. Despite symptomatic treatment, the symptoms persisted and progressed into severe pain in the right leg and an inability to bear weight, and he was referred to our hospital due to photophobia, neck rigidity, and hypoactivity, which raised suspicion for meningitis. Brain CT and lumbar puncture were unremarkable. The patient was admitted for further evaluation and started on empirical antibiotics (vancomycin and ceftriaxone).

**FIGURE 1:**
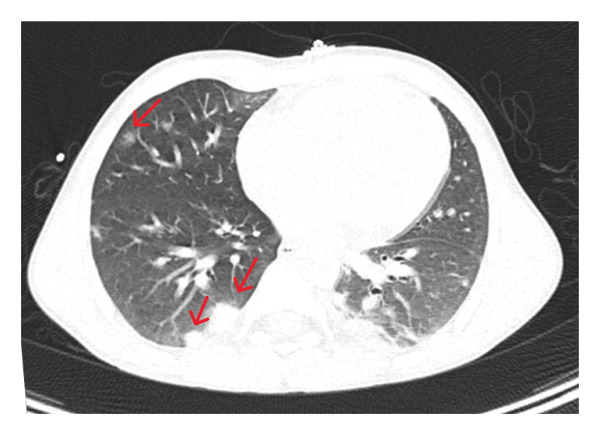
Chest CT pulmonary angiogram demonstrating multiple bilateral patchy, dense consolidations, most prominent in the lower lobes, consistent with septic emboli.

**FIGURE 2:**
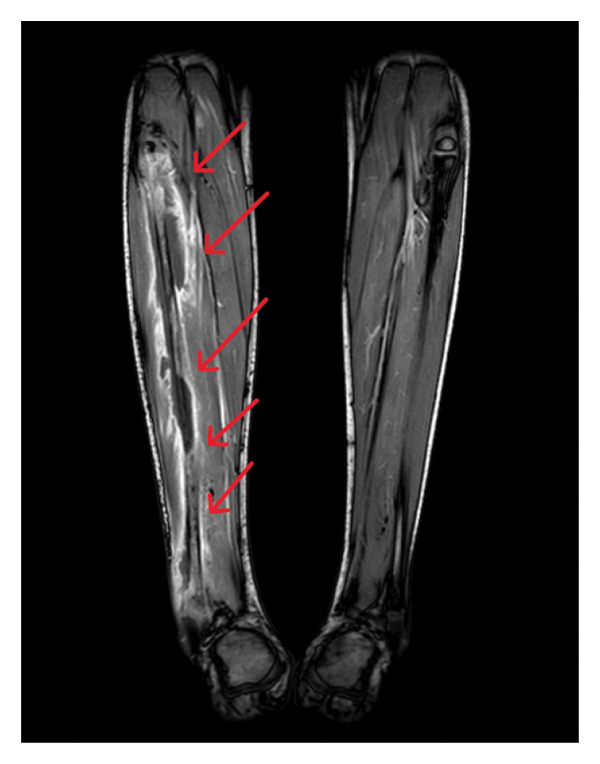
MRI of the lower limbs (T1‐weighted postcontrast) demonstrating a multiloculated rim‐enhancing fluid collection surrounding the right fibular diaphysis, extending craniocaudally for approximately 27 cm with a maximal thickness of 1.5 cm. Associated periosteal elevation with reactive periosteal enhancement is noted. Subtle cortical erosions are present along the medial and posterior aspects of the proximal fibular metaphysis.

On admission, the patient was febrile, complaining of chest pain, left forearm pain, photophobia, and neck rigidity. He appeared ill but was alert and oriented (GCS 15/15). Vital signs were stable. Physical examination revealed swelling and tenderness in the left forearm and right calf, with no open wounds or punctures. No neurological deficits were noted. Laboratory tests showed elevated CRP (269), ESR (95), mild anemia (Hb: 10.2), and thrombocytopenia (PLT: 122). D‐dimer was significantly elevated (16.6). Diagnostic imaging including chest CT angiography showed a filling defect in the left upper segmental pulmonary artery, consistent with segmental pulmonary embolism (PE), multiple bilateral patchy consolidations (septic emboli) (Figure 1), and minimal pleural effusion. Abdominal ultrasound was done and revealed mild hepatosplenomegaly with normal echotexture. There was a mild amount of pelvic free fluid. X‐rays showed no fractures. Doppler ultrasound of the left arm revealed DVT in the brachial and cephalic veins, as well as superficial thrombophlebitis. No DVT was found in the right leg. MRI suggested osteomyelitis in the right fibula with a subperiosteal abscess. MRI findings showed a multiloculated fluid collection surrounding the right fibular diaphysis, with associated cortical erosion, suggestive of osteomyelitis. There was also a thrombus in the right posterior tibial vein (Figure 2).

Blood cultures identified MRSA. The patient was initially treated with vancomycin, ceftriaxone, acyclovir, and doxycycline, with a diagnosis of toxic shock syndrome; after the cultures, the antibiotic coverage was narrowed to vancomycin and trimethoprim–sulfamethoxazole. The overall duration of antibiotics was approximately 5 weeks (in and outpatient therapy). Anticoagulation with a therapeutic dose of LMWH (enoxaparin) was also started, with an overall duration of 14 days (in‐hospital) and continued for 3 months after discharge. Over the next several days, the patient’s condition improved significantly. Swelling in the extremities reduced, and he regained the ability to move both the right leg and left forearm with minimal limitation. The fever resolved, and the patient’s CRP decreased to normal levels (CRP: 6). The right leg abscess was drained, and no new collections were noted. The patient’s laboratory values normalized, and he showed significant functional improvement. The patient was discharged in a vitally stable condition; he was afebrile, active, with full range of motion in both limbs and no signs of deformity.

### 2.2. Case #2

**FIGURE 3:**
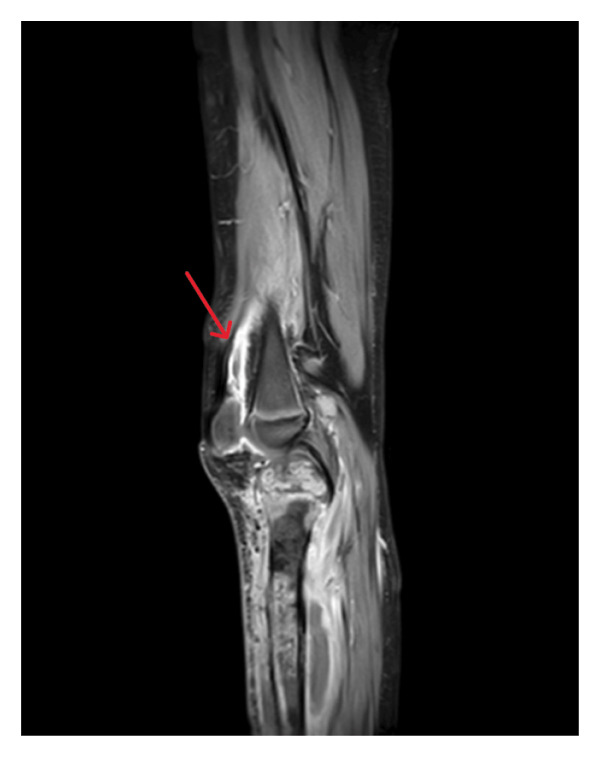
MRI of the left knee (T1‐weighted postcontrast) demonstrating a suprapatellar fluid collection with a thick, enhancing wall, consistent with an abscess.

**FIGURE 4:**
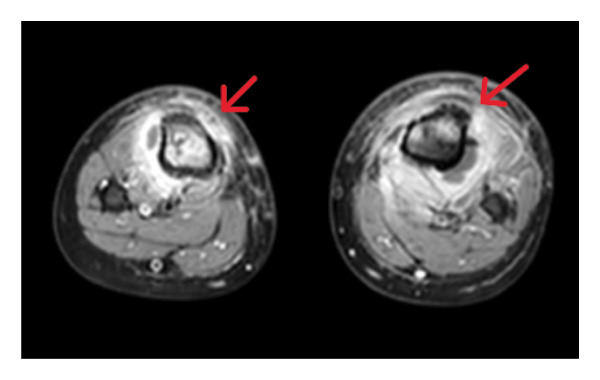
MRI of the lower limbs (T1‐weighted postcontrast) demonstrating multiple areas of low signal intensity with heterogeneous postcontrast enhancement involving the epiphysis and metaphysis of both proximal tibiae. The surrounding muscle compartments of both legs show heterogeneous edema with associated enhancement.

An 8‐year‐old male, previously healthy and fully immunized, presented with lower limb pain, hypoactivity, recurrent vomiting, and respiratory distress, including tachypnea, following a fall. His family initially sought medical attention at an outside hospital, where several diagnostic tests were conducted. A chest CT angiography revealed multiple septic emboli in the lungs, and the patient was promptly started on LMWH (enoxaparin) for anticoagulation. Doppler ultrasound revealed superficial thrombophlebitis with echogenic content in the right short saphenous vein, along with inflammatory changes along the tibia. Blood flow in the deep femoral veins was normal. An abdominal ultrasound and brain CT, performed due to the history of the fall, showed no abnormalities. Laboratory tests indicated elevated CRP (444) and ESR (80). Blood cultures were positive for MRSA, and the patient was started on a combination of vancomycin and ceftriaxone. He was subsequently transferred to our hospital for further evaluation and management of bilateral lower limb cellulitis and sepsis. On examination, the patient was febrile with redness, warmth, and swelling in both lower limbs, accompanied by limited movement. Further laboratory tests revealed elevated D‐dimer levels (6.85), suggesting a thrombotic event. A lower limb MRI demonstrated osteomyelitis in both tibiae, with the right tibia being more severely affected. The MRI findings were consistent with osteomyelitis (Figures 3 and 4).

Management included IV vancomycin, adjusted based on drug levels, clindamycin, and continued anticoagulation with enoxaparin. The overall duration of antibiotics and anticoagulation was approximately 7 weeks (in and outpatient). As the patient’s condition began to improve, he developed right‐sided abdominal pain. An abdominal ultrasound revealed mild enlargement of the liver, spleen, and kidneys, with no other significant abnormalities. The patient underwent ultrasound‐guided aspiration by interventional radiology from both lower limbs, with a right leg drain placed. Fluid analysis revealed a bloody appearance, high LDH (39,700), and elevated white blood cells (236,000). The fluid cultures confirmed the presence of MRSA. Two subsequent ultrasound‐guided aspirations were performed. Despite persistent fever, the patient’s clinical condition stabilized, and his CRP levels began to improve (194). After a few days, there was a noticeable reduction in swelling and tenderness in the legs. The drains were removed as the output decreased, and CRP continued to decrease further to 118. The patient’s recovery was supported by ongoing IV antibiotics and physiotherapy for his legs. A repeat MRI of the lower limbs showed stable findings, with no new significant collections. One week later, with continued therapy, the patient was discharged in a vitally stable condition. He was afebrile, active, with a full range of motion in both lower limbs and no signs of deformity.

## 3. Discussion and Review of Literature

Pediatric cases presenting with the combined triad of acute osteomyelitis, DVT, and SPE are exceedingly rare, with fewer than fifteen well‐documented reports in the literature (Table 1). Most published cases involve school‐aged children and adolescents, typically following hematogenous osteomyelitis of long bones most commonly the femur, tibia, fibula, or hip, although upper‐extremity and scapular involvement have also been described. Reported vascular complications frequently include thrombosis of major proximal limb veins, and the septic emboli almost invariably manifest as pulmonary nodules or cavitating lesions on chest CT. *S. Aureus* remains the predominant pathogen, including both MSSA and community‐acquired MRSA (CA‐MRSA); several reports specifically note Panton–Valentine leucocidin (PVL)–producing strains. Less commonly, nonstaphylococcal organisms such as ESBL‐producing *Klebsiella pneumoniae* have been implicated [3–12].

**TABLE 1:** Summary of reported pediatric cases of osteomyelitis with deep vein thrombosis and septic pulmonary emboli.

	1	2	3	4	5	6	7	8	9	10	11	12	13	14
Author/Year	Yuksel et al. (2004) [5]	LePage et al. (2008) [3]	LePage et al. (2008) [3]	Kuhfahl et al. (2009) [6]	Obando et al. (2011) [4]	Obando et al. (2011) [4]	Schaub and Rodkey (2012) [7]	Sheikh Najeeb et al. (2023) [8]	Harda et al. (2023) [9]	Chaar et al. (2025) [10]	Pena et al. (2025) [11]	Khurtsilava et al. (2015) [12]	Case 1	Case 2
Age (years)	3	13	15	10	12	7	5	16	10	9	14	13	11	8
Sex	M	M	M	M	F	F	M	M	M	M	M	M	M	M
Precipitating event/factor	—	Minor trauma (wrestling)	Minor trauma (fall)	Minor trauma (fall)	Minor trauma	—	Minor trauma (fall)	Minor trauma (fall)	—	Minor trauma (fall)	Minor trauma (fall)	Minor trauma (scrapping)	Minor trauma	Minor trauma (fall)
Osteomyelitis site	Right distal fibula	Lateral aspect of the proximal left humerus	Intertrochanteric region of the left femur	Left anterior and posterior scapula.	Left tibia	Right femur	Midshaft left tibia	Left hip joint (femoral neck)	Right ilium	Left thigh, knee, and coxofemoral joint	Proximal left tibia	Proximal left tibia	Right fibula	Right and left tibiae
Thrombosis site	Right external iliac vein, extended to the popliteal vein	Cephalic vein	Left common femoral, Deep popliteal and distal Common iliac veins.	Left subclavian, axillary, and brachial veins (occlusive), left innominate and azygous veins (nonocclusive)	Deep and superficial femoral veins	N/R	Left popliteal and distal superficial femoral veins.	Left femoral vein.	Right common iliac vein.	Left common femoral vein.	Left femoral and popliteal veins.	N/S	Left brachial, Cephalic and right posterior tibial veins	Right short saphenous vein.
Septic emboli	Pulmonary	Pulmonary	Pulmonary	Pulmonary	Pulmonary	—	Pulmonary	Pulmonary	Pulmonary	Pulmonary	Pulmonary	Pulmonary	Pulmonary	Pulmonary
Isolated pathogen	*S. aureus*	MSSA	*Fusobacterium necrophorum*	CA‐MRSA	PVL‐positive MSSA	PVL‐positive MSSA	MRSA	*Klebsiella pneumoniae* (fluoroquinolone‐sensitive ESBL strain)	PVL‐positive MRSA	MSSA	MSSA	N/S	MRSA	MRSA
Hepatosplenomegaly	—	Present	—	—	—	—	—	—	—	—	—	Present	Present	Present
Treatment														
Anticoagulation	LMWH (Enoxaparin)	LMWH (Enoxaparin)	N/S anticoagulant	LMWH (Enoxaparin)	N/S anticoagulant	N/S anticoagulant	LMWH (Enoxaparin)	LMWH (Enoxaparin)	N/R	LMWH (enoxaparin)	LMWH (enoxaparin)	LMWH (enoxaparin)	Low molecular weight heparin (enoxaparin)	LMWH (enoxaparin)
Antibiotics	Vancomycin and amikacin	N/S antibiotics	N/S antibiotics	Gentamicin, vancomycin, and TMP/SMX	Cloxacillin, clindamycin, and cefadroxil	Cloxacillin and clindamycin	Clindamycin	Meropenem	Linezolid, vancomycin, and clindamycin	Vancomycin, and piperacillin–tazobactam	Vancomycin, linezolid, cloxacillin, and ceftaroline	Vancomycin and cefepime	Vancomycin and TMP/SMX	Vancomycin and clindamycin
Surgical intervention	Surgical drainage	Surgical drainage	surgical irrigation and debridement	N/R	Surgical irrigation	N/R	Surgical drainage	Surgical drainage	Surgical drainage	Surgical drainage	IVC filter and surgical debridement	Surgical drainage	Surgical drainage	Multiple ultrasound‐guided aspiration
Outcomes	Recovered	Recovered	Recovered	Recovered	Recovered	Recovered	Recovered	Joint stiffness and decreased mobility leading to hip joint replacement.	Recovered	Recovered	Recovered	Recovered	Recovered	Recovered

The typical clinical sequence described in the literature begins with minor nonpenetrating trauma followed by hematogenous osteomyelitis, progression to septic thrombophlebitis, and subsequent showering of septic emboli. Early phases are often clinically nonspecific with fever, localized limb pain, limp, or soft‐tissue swelling, leading clinicians to initially consider musculoskeletal trauma, viral illness, or uncomplicated cellulitis. This overlap in presentation contributes to diagnostic delay, particularly because DVT and PE are uncommon in otherwise healthy children and may not be immediately suspected. In many cases, initial radiographic evaluations are unrevealing, and the diagnosis becomes clearer only after the onset of respiratory symptoms or persistent systemic inflammation prompting advanced imaging.

The two cases described here share many features with previously reported presentations but also display an unusual clinical sequence. Both children were previously healthy, experienced minor trauma, and presented with limb pain, fever, and significantly elevated inflammatory markers. However, each developed PE or septic emboli early in the disease course before or concurrent with the confirmatory diagnosis of osteomyelitis. This pattern suggests a highly aggressive infection related to MRSA bacteremia, in which septic venous thrombosis or embolic seeding may have served as the nidus for subsequent multifocal bone involvement. The multifocal osteomyelitis observed in both patients, with right fibular involvement in Case 1 and bilateral tibial involvement in Case 2, supports a hematogenous mechanism rather than localized contiguous spread.

Initial imaging studies were nondiagnostic in both children, a finding consistent with early literature describing the limited sensitivity of plain radiographs and early CT scans in acute osteomyelitis. MRI ultimately proved essential in identifying multifocal marrow involvement, subperiosteal abscesses, cortical disruption, and deep soft‐tissue collections. These findings underscore the importance of early MRI in febrile children with persistent limb pain, systemic inflammatory response, and markedly elevated CRP or D‐dimer, particularly in the setting of confirmed MRSA bacteremia or unexplained DVT/PE.

Management in both cases required aggressive, multidisciplinary coordination, reflecting the complexity described in prior reports. Therapy included early broad‐spectrum intravenous antibiotics followed by targeted MRSA coverage, therapeutic anticoagulation in the presence of venous thrombosis or septic emboli, and surgical drainage of deep collections. Drainage was especially critical in Case 2, where recurrent abscess formation required repeated ultrasound‐guided aspiration and extended antimicrobial therapy. Both patients ultimately recovered; however, the second case demonstrated a more prolonged and fluctuating clinical course, consistent with the more extensive bilateral disease burden.

Collectively, these cases reinforce that osteomyelitis in children may develop as a complication of septic emboli or septic venous thrombosis in the setting of MRSA bacteremia rather than solely as a precursor to thromboembolic disease. Recognizing this bidirectional relationship is critical to avoid delays in diagnosis. Clinicians should maintain a high index of suspicion for musculoskeletal infection in pediatric patients who present with persistent limb pain and systemic inflammation following PE/DVT or in the context of documented MRSA bacteremia. Early MRI, close monitoring of inflammatory markers, and prompt multidisciplinary management are essential to minimizing morbidity and preventing long‐term sequelae. A limitation of this report is the lack of comprehensive follow‐up data, including posttreatment diagnostic imaging and laboratory investigations.

## 4. Conclusion

Osteomyelitis complicated by DVT and SPE is a rare but serious presentation in children. These two cases illustrate that multifocal osteomyelitis may result from septic thrombosis or embolic seeding in the setting of MRSA bacteremia, often preceding clear radiologic evidence of bone infection. Because early symptoms are nonspecific and initial imaging may be unrevealing, clinicians should maintain suspicion for musculoskeletal infection in pediatric patients with persistent limb pain, elevated inflammatory markers, or unexplained thromboembolic events. Early MRI, targeted antimicrobial therapy, anticoagulation when indicated, and coordinated surgical management are essential to reducing morbidity and achieving full recovery.

**Nomenclature**
DVT: Deep vein thrombosis
PSE: Pulmonary septic emboli
M: Male
F: Female
*S. Aureus*
: *Staphylococcus aureus*
MSSA: Methicillin‐sensitive *Staphylococcus aureus*
MRSA: Methicillin‐resistant *Staphylococcus aureus*
CA‐MRSA: Community acquired MRSA
PVL: Panton–Valentine leucocidin
ESBL: Extended‐spectrum beta‐lactamase
LMWH: Low molecular weight heparin
TMP/SMX: Trimethoprim–sulfamethoxazole

## Author Contributions

All authors contributed to the study design, manuscript writing, figure preparation, editing, and critical revision.

## Funding

This research received no external funding.

## Ethics Statement

The study was conducted in accordance with ethical standards.

## Consent

Informed consent for publication was obtained.

## Conflicts of Interest

The authors declare no conflicts of interest.

## Data Availability

The dataset generated and/or analyzed during the current study are available from the corresponding author upon reasonable request.
